# A guide to selecting high-performing antibodies for Huntingtin (UniProt ID: P42858) for use in western blot, immunoprecipitation, and immunofluorescence

**DOI:** 10.12688/f1000research.153670.2

**Published:** 2025-01-02

**Authors:** Rebeka Fanti, Riham Ayoubi, Charles Alende, Maryam Fotouhi, Sara González Bolívar, Renu Chandrasekaran, Kathleen Southern, Aled M. Edwards, Rachel J. Harding, Carl Laflamme, NeuroSGC/YCharOS/EDDU collaborative group, ABIF consortium

**Affiliations:** 1 Structural Genomics Consortium, University of Toronto, Toronto, Ontario, Canada; 2 Department of Neurology and Neurosurgery, Structural Genomics Consortium, The Montreal Neurological Institute, McGill University, Montreal, Québec, Canada; 3 Department of Pharmacology and Toxicology, University of Toronto, Toronto, Ontario, Canada

**Keywords:** UniProt ID P42858, HTT, Huntingtin, antibody characterization, antibody validation, western blot, immunoprecipitation, immunofluorescence

## Abstract

Huntingtin encodes a 3144 amino acid protein, with a polyglutamine repeat tract at the N-terminus. Expansion of this repeat tract above a pathogenic threshold of 36 repeats is the causative mutation of Huntington's disease, a neurodegenerative disorder characterized by loss of striatal neurons. Here we have characterized twenty Huntingtin commercial antibodies for western blot, immunoprecipitation, and immunofluorescence using a standardized experimental protocol based on comparing read-outs in knockout cell lines and isogenic parental controls. These studies are part of a larger, collaborative initiative seeking to address antibody reproducibility issues by characterizing commercially available antibodies for human proteins and publishing the results openly as a resource for the scientific community. While use of antibodies and protocols vary between laboratories, we encourage readers to use this report as a guide to select the most appropriate antibodies for their specific needs.

## Introduction

Huntington’s Disease (HD) is a neurodegenerative disorder inherited in an autosomal dominant manner, presenting with a spectrum of progressive motor, cognitive, and psychological impairments, typically with adult-onset of symptoms. ^1^ Although the HD causative gene, *HTT*, was discovered over three decades ago, there are still no disease-modifying treatments available for patients, and progress unpicking the molecular pathology of the disease remains slow. ^2^

HD arises from a heterozygous expansion mutation of the trinucleotide CAG repeat tract in exon 1 of *HTT*, located on chromosome 4, above a critical threshold of ~36 repeats. This mutation results in expansion of the polyglutamine stretch at the N-terminus of the 3144 amino acid Huntingtin protein. Huntingtin functions as a scaffold protein, engaging in extensive protein-protein interactions, ^3^ forming various multi-protein complexes to carry-out its diverse array of functions. Modulation of this interaction network by the polyglutamine expansion contributes to degeneration within the central nervous system, affecting medium spiny neurons at the onset of disease. ^4^^,^^5^

The low expression level, complex interactome and large size of the 348 kDa Huntingtin protein have given rise to technical challenges which have hindered precise determination of its molecular function, or how this is altered in disease. In particular, the use of different Huntingtin antibodies by scientists in the HD research community, often mapping to structurally distant epitopes, can yield different or even conflicting results, further conflating interrogation of this protein. ^6^^–^^8^

This research is part of a broader collaborative initiative in which academics, funders and commercial antibody manufacturers are working together to address antibody reproducibility issues by characterizing commercial antibodies for human proteins using standardized protocols, and openly sharing the data. ^9^^–^^11^ Here we evaluated the performance of twenty commercial antibodies for Huntingtin for use in western blot, immunoprecipitation, and immunofluorescence, enabling biochemical and cellular assessment of Huntingtin properties and function. The platform for antibody characterization used to carry out this study was endorsed by a committee of industry and academic representatives. It consists of identifying human cell lines with adequate target protein expression and the development/contribution of equivalent knockout (KO) cell lines, followed by antibody characterization procedures using most commercially available antibodies against the corresponding target protein. The standardized consensus antibody characterization protocols are openly available on Protocol Exchange (DOI: 10.21203/rs.3.pex-2607/v1). ^12^

The authors do not engage in result analysis or offer explicit antibody recommendations. A limitation of this study is the use of universal protocols - any conclusions remain relevant within the confines of the experimental setup and cell line used in this study. Our primary aim is to deliver top-tier data to the scientific community, grounded in Open Science principles. This empowers experts to interpret the characterization data independently, enabling them to make informed choices regarding the most suitable antibodies for their specific experimental needs. Guidelines on how to interpret antibody characterization data found in this study are featured on the YCharOS gateway. ^13^

## Results and discussion

**Figure 1.**
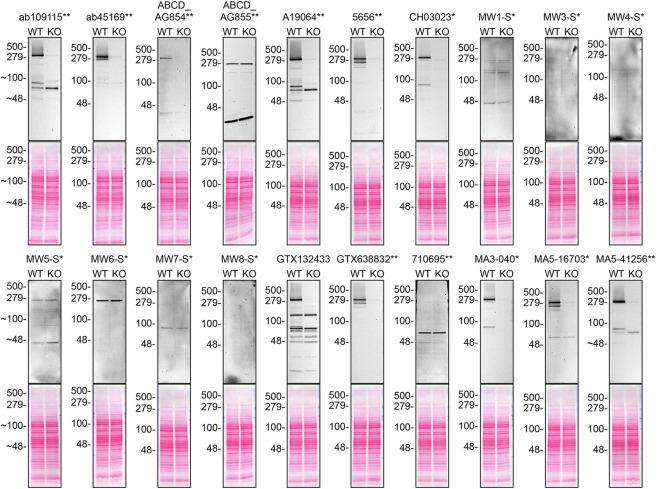
Huntingtin antibody screening by western blot. Lysates of DMS 53 (WT and *HTT* KO) were prepared and 30 μg of protein were processed for western blot with the indicated Huntingtin antibodies. Tris-Glycine 4-20% gels were used for SDS-PAGE. The Ponceau stained transfers of each blot are presented to show equal loading of WT and KO lysates and protein transfer efficiency from the acrylamide gels to the nitrocellulose membrane. Antibody dilutions were chosen according to the recommendations of the antibody supplier. Exceptions were given for antibodies ab109115**, ab45169**, and MA5-41256** which were titrated as the signal was too weak when following the supplier’s recommendations. Antibody dilution used: ab109115** at 1/2000, ab45169** at 1/5000, ABCD_AG854** at 1/10, ABCD_AG855** at 1/10, A19064** at 1/1000, 5656** at 1/1000, CH03023* at 1/1000, MW1-S* at 1/10, MW3-S* at 1/10, MW4-S* at 1/10, MW5-S* at 1/10, MW6-S* at 1/10, MW7-S* at 1/10, MW8-S* at 1/10, GTX132433 at 1/500, GTX638832** at 1/500, 710695** at 1/200, MA3-040* at 1/1000, MA5-16703* at 1/500, MA5-41256** at 1/2000. Predicted band size: 347 kDa. *Monoclonal antibody, **Recombinant antibody. Note: MW1-S*, MW3-S*, MW4-S*, MW5-S*, MW6-S*, MW7-S* and MW8-S* are expected to only recognize an altered conformation of the polyQ domain generated as the polyQ domain of Huntingtin increases in length.

**Figure 2.**
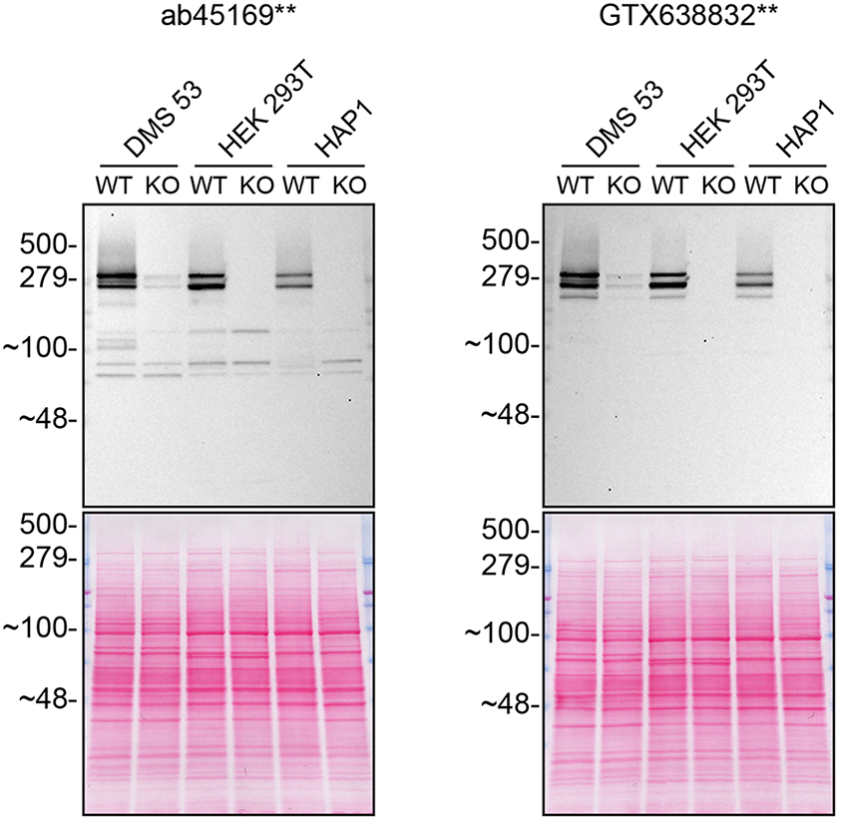
Huntingtin western blot on various cell lysates. Lysates of WT and *HTT* KO in DMS 53, HEK 293T and HAP1 were prepared, and 30 μg of protein was processed for western blot with the indicated Huntingtin antibodies ab45169** at 1/5000 and GTX638832** at 1/500. Tris-Glycine 4-20% gels were used for SDS-PAGE. The Ponceau stained transfer is shown as a loading control. Predicted band size: 347 kDa. **Recombinant antibody.

Our standard protocol involves comparing readouts from WT (wild type) and KO cells. ^14^^,^^15^ The first step is to identify a cell line(s) that expresses sufficient levels of a given protein to generate a measurable signal. To this end, we examined the DepMap transcriptomics database to identify all cell lines that express the target at levels greater than 2.5 log _2_ (transcripts per million “TPM” + 1), which we have found to be a suitable cut-off (Cancer Dependency Map Portal, RRID:SCR_017655). We generated an *HTT* KO line in DMS 53 as it expresses the endogenous *HTT* transcript at 6.1 log _2_ (TPM+1), which is above the average range of cancer cell lines analyzed. A commercial HAP1 *HTT* KO is also available; HAP1 expresses *HTT* at 3.7 log _2_ (TPM+1) RNA level. A HEK293T *HTT* KO cell line has been developed and used elsewhere ^16^ ( Table 1). All three cell line backgrounds were evaluated by western blot using a high-performing Huntingtin antibody detected in Figure 1. DMS 53 was identified as the most suitable cell line ( Figure 2), which can be explained by its high expression of the *HTT* transcript compared to other two cell lines. Thus, DMS 53 WT and KO cell lines were generated and used to evaluate the antibodies in all applications.

**Table 1.** Summary of the cell lines used.

Institution	Catalog number	RRID (Cellosaurus)	Cell line	Genotype
ATCC	CRL-2062	CVCL_1177	DMS 53	WT
Academic	non-commercial	CVCL_D6U0	DMS 53	*HTT* KO
ATCC	CRL-3216	CVCL_0063	HEK 293T	WT
Academic	non-commercial	CVCL_D7EP	HEK 293T	*HTT* KO ^16^
Horizon Discovery	C631	CVCL_Y019	HAP1	WT
Horizon Discovery	HZGHC004595c006	CVCL_SR86	HAP1	*HTT* KO

For western blot experiments, WT and *HTT* KO protein lysates were ran on SDS-PAGE, transferred onto nitrocellulose membranes, and then probed with twenty Huntingtin antibodies in parallel ( Table 2, Figure 1).

**Table 2.** Summary of the Huntingtin antibodies tested.

Company	Catalog number	Lot number	RRID (Antibody Registry)	Clonality	Clone ID	Host	Concentration (μg/μl)	Vendors recommended applications
Abcam	ab109115 **	1003147-4	AB_10863082	recombinant-mono	EPR5526	rabbit	1.52	Wb, IF
Abcam	ab45169 **	1022500	AB_733062	recombinant-mono	EP867Y	rabbit	1.55	Wb, IF
ABCD Antibodies	ABCD_AG854 **	10/27/2023	AB_3076339	recombinant-mono	12.3	rabbit	0.004	n/a
ABCD Antibodies	ABCD_AG855 **	10/27/2023	AB_3076340	recombinant-mono	C4	rabbit	0.19	n/a
ABclonal	A19064 **	4000000431	AB_2862557	recombinant-mono	ARC0431	rabbit	0.63	Wb, IF
Cell Signaling Technology	5656 **	6	AB_10827977	recombinant-mono	D7F7	rabbit	0.10	Wb, IF
Coriell Institute	CH03023 *	03.17.21	AB_3096092	monoclonal	2B7	mouse	1.71	n/a
DSHB	MW1-S *	44322	AB_528290	monoclonal	MW1-S	mouse	0.02	Wb, IP, IF
DSHB	MW3-S *	43216	AB_528292	monoclonal	MW3-S	mouse	0.01	Wb, IF
DSHB	MW4-S *	43251	AB_528293	monoclonal	MW4-S	mouse	0.02	Wb, IF
DSHB	MW5-S *	44742	AB_528294	monoclonal	MW5-S	mouse	0.03	Wb, IF
DSHB	MW6-S *	43230	AB_528295	monoclonal	MW6-S	mouse	0.06	Wb, IF
DSHB	MW7-S *	43461	AB_528296	monoclonal	MW7-S	mouse	0.03	Wb, IF
DSHB	MW8-S *	44315	AB_528297	monoclonal	MW8-S	mouse	0.04	Wb, IP, IF
GeneTex	GTX132433	42312	AB_2886646	polyclonal	MW3-S	rabbit	1.40	Wb, IF
GeneTex	GTX638832 **	45096	AB_3094813	recombinant-mono	HL2483	rabbit	0.98	Wb
Thermo Fisher Scientific	710695 **	RF236710	AB_2608784	recombinant-poly	3HCLC	rabbit	0.50	IF
Thermo Fisher Scientific	MA3-040 *	YG376237	AB_2608783	monoclonal	1HU-4C8	mouse	n/a	Wb, IF
Thermo Fisher Scientific	MA5-16703 *	YE3913821A	AB_2538195	monoclonal	HDB4E10	mouse	1.00	Wb, IP, IF
Thermo Fisher Scientific	MA5-41256 **	YE3913382B	AB_2899009	recombinant-mono	JB89-34	rabbit	1.00	Wb, IF

Wb=western blot; IF=immunofluorescence; IP=immunoprecipitation; n/a=not available, DSHB=Developmental Studies Hybridoma Bank.

* Monoclonal antibody,

** Recombinant antibody.

**Figure 3.**
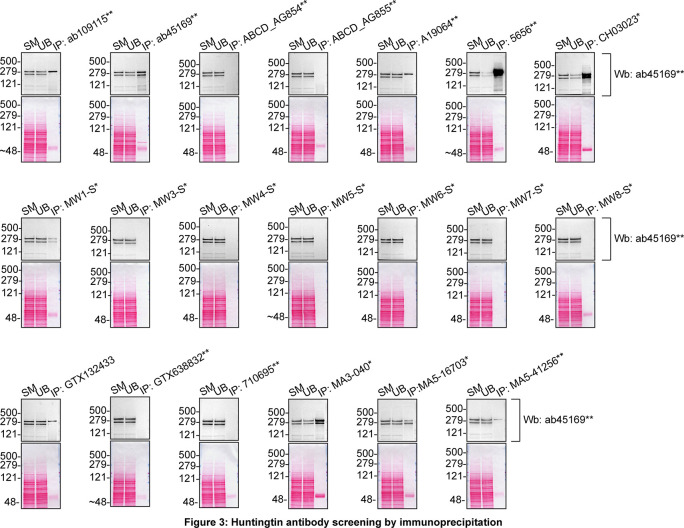
Huntingtin antibody screening by immunoprecipitation. DMS 53 lysates were prepared, and immunoprecipitation was performed using 2.0 μg of the indicated Huntingtin antibodies pre-coupled to Dynabeads protein A or protein G. The concentration of MA3-040* is unknown and therefore 5 μL of this antibody was tested. All samples were washed and processed for western blot with the indicated Huntingtin antibody. Tris-Glycine 4-20% gels were used for SDS-PAGE. For western blot, ab45169** was used at 1/5000. The Ponceau stained transfers of each blot are shown. SM=4% starting material; UB=4% unbound fraction; IP=immunoprecipitate. *Monoclonal antibody, **Recombinant antibody.

We then assessed the capability of all twenty antibodies to capture Huntingtin from DMS 53 protein extracts using immunoprecipitation techniques, followed by western blot analysis. For the immunoblot step, a specific Huntingtin antibody identified previously (refer to Figure 1) was selected. Equal amounts of the starting material (SM), the unbound fraction (UB), as well as the whole immunoprecipitate (IP) eluates were separated by SDS-PAGE ( Figure 3).

**Figure 4.**
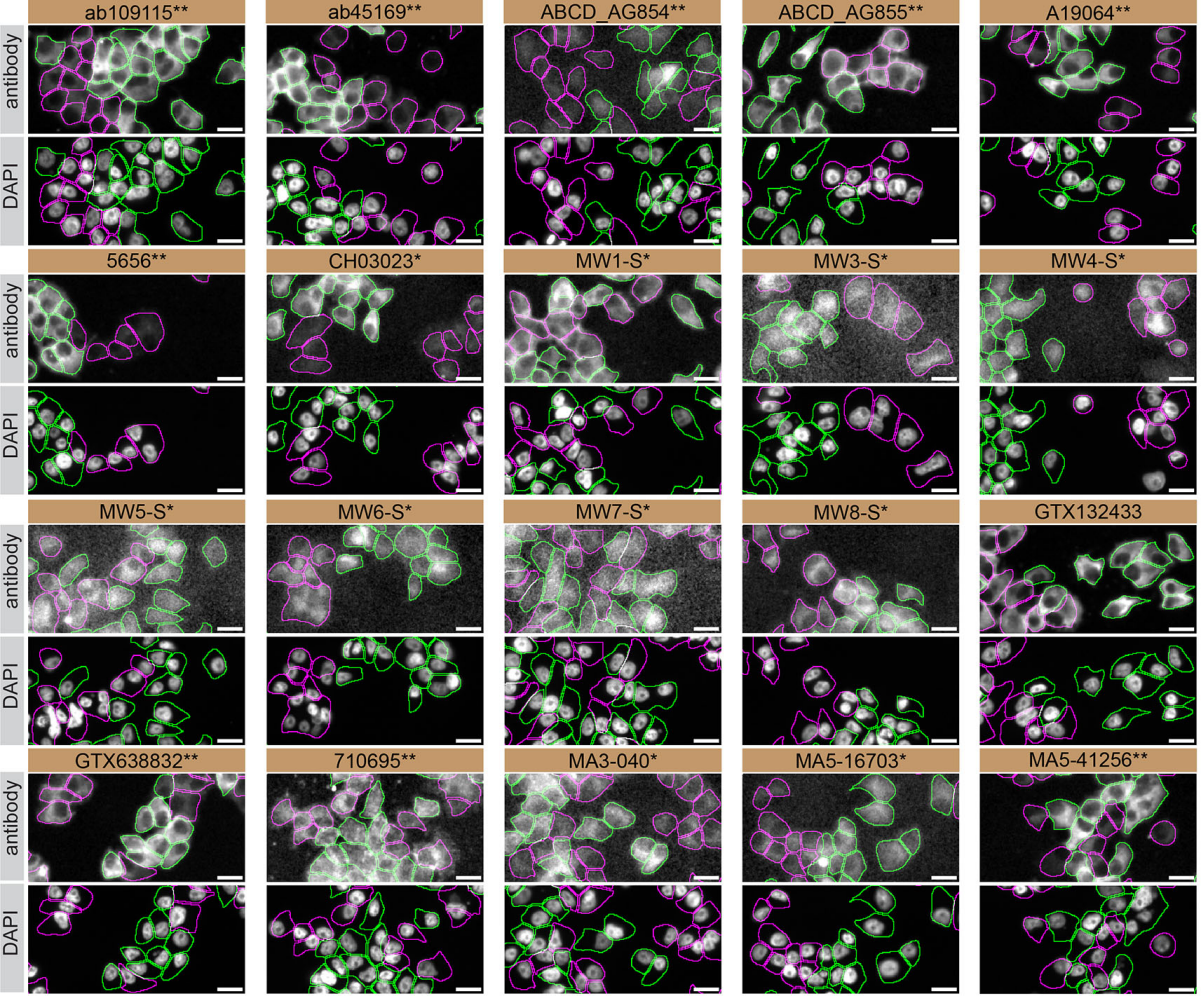
Huntingtin antibody screening by immunofluorescence. DMS 53 WT and *HTT* KO cells were labelled with a green or a far-red fluorescent dye, respectively. WT and KO cells were mixed and plated to a 1:1 ratio on coverslips. Cells were stained with the indicated Huntingtin antibodies and with the corresponding Alexa-fluor 555 coupled secondary antibody including DAPI. Acquisition of the blue (nucleus-DAPI), green (WT), red (antibody staining) and far-red (KO) channels was performed. Representative images of the merged blue and red (grayscale) channels are shown. WT and KO cells are outlined with green and magenta dashed line, respectively. When an antibody was recommended for immunofluorescence by the supplier, we tested it at the recommended dilution and at 1 μg/ml. The rest of the antibodies were tested at 1 and 2 μg/ml. The final concentration of each antibody was selected based on the detection range of the microscope used and a quantitative analysis not shown here. Antibody dilutions corresponding to the images shown are: ab109115** at 1/1500, ab45169** at 1/1500, ABCD_AG854** at 1/500, ABCD_AG855** at 1/200, A19064** at 1/600, 5656** at 1/100, CH03023* at 1/1700, MW1-S* at 1/20, MW3-S* at 1/10, MW4-S* at 1/10, MW5-S* at 1/15, MW6-S* at 1/60, MW7-S* at 1/15, MW8-S* at 1/20, GTX132433 at 1/500, GTX638832** at 1/500, 710695** at 1/500, MA3-040* at 1/1000, MA5-16703* at 1/1000, MA5-41256** at 1/1000. Bars = 10 μm. *Monoclonal antibody, **Recombinant antibody.

For immunofluorescence, twenty antibodies were screened using a mosaic strategy. First, DMS 53 WT and *HTT* KO cells were labelled with different fluorescent dyes in order to distinguish the two cell lines, and the Huntingtin antibodies were evaluated. Both WT and KO lines were imaged in the same field of view to reduce staining, imaging and image analysis bias ( Figure 4). Quantification of immunofluorescence intensity in hundreds of WT and KO cells was performed for each antibody tested, ^12^ and the images presented in Figure 3 are representative of this analysis.

In conclusion, we have screened twenty commercial Huntingtin antibodies by western blot, immunoprecipitation, and immunofluorescence by comparing the signal produced by the antibodies in human DMS 53 WT and *HTT* KO cells. Several high-quality and renewable Huntingtin antibodies were identified in all applications. Researchers who wish to study Huntingtin in a different species are encouraged to select high-quality antibodies, based on the results of this study, and investigate the predicted species reactivity of the manufacturer before extending their research.

The underlying data for this study can be found on Zenodo, an open-access repository for which YCharOS has its own collection of antibody characterization reports. ^17^

## Methods

The standardized protocols used to carry out this KO cell line-based antibody characterization platform was established and approved by a collaborative group of academics, industry researchers and antibody manufacturers. The detailed materials and step-by-step protocols used to characterize antibodies in western blot, immunoprecipitation and immunofluorescence are openly available on Protocol Exchange, a preprint server (DOI: 10.21203/rs.3.pex-2607/v1). ^12^

### Antibodies and cell lines used

Cell lines used and primary antibodies tested in this study are listed in Tables 1 and 2, respectively. To ensure that the cell lines and antibodies are cited properly and can be easily identified, we have included their corresponding Research Resource Identifiers, or RRID. ^18^^,^^19^

## Data Availability

Zenodo: Dataset for the Huntingtin antibody screening study, doi.org/10.5281/zenodo.11639052. ^17^ Data are available under the terms of the Creative Commons Attribution 4.0 International license (CC-BY 4.0).
