# Supplementary material for: A guide to selecting high-performing antibodies for Huntingtin (UniProt ID: P42858) for use in western blot, immunoprecipitation, and immunofluorescence
Source: F1000Res. 2025 Jan 2;13:922. Originally published 2024 Aug 13. [Version 2] doi: 10.12688/f1000research.153670.2 (PMC11384196; doi:10.12688/f1000research.153670.2)
Supplement: Peer review history

# Reviewer report: Reviewer response for version 2

**DOI:** 10.5256/f1000research.176211.r360581

**Published:** 2025-02-12

**Authors:** Daniele Bertoglio

**Affiliations:** 1 University of Antwerp, Antwerp, Belgium

This manuscript by Fanti et al. presents a comprehensive evaluation of twenty commercially available HTT antibodies utilizing standardized experimental protocols. The study systematically assesses the suitability and sensitivity of these antibodies in Western blot, immunoprecipitation, and immunofluorescence applications, employing both wild-type and knock-out HTT cell lines. It would have been of additional support inclusion of immunohistochemistry, but this does not restrict the value of the work.

 The study addresses a critical technical challenge in Huntington’s Disease (HD) research, namely the variability in antibody performance, which has posed a significant issue within the field.

 The experimental approach is well conceived, designed, and executed. The data are presented in a clear and accessible manner, facilitating ease of interpretation. The findings provide valuable guidance for researchers seeking to source and utilize specific HTT antibodies in their investigations. Overall, this study constitutes a significant contribution to the HD research community, offering essential data to improve experimental consistency and reproducibility

Are sufficient details of methods and materials provided to allow replication by others?

Yes

Is the rationale for creating the dataset(s) clearly described?

Yes

Are the datasets clearly presented in a useable and accessible format?

Yes

Are the protocols appropriate and is the work technically sound?

Yes

Reviewer Expertise:

In vivo and post-mortem imaging of neurodegenetive disorders, including HD

I confirm that I have read this submission and believe that I have an appropriate level of expertise to confirm that it is of an acceptable scientific standard.

# Reviewer report: Reviewer response for version 2

**DOI:** 10.5256/f1000research.176211.r358661

**Published:** 2025-02-06

**Authors:** Srinivasa Subramaniam

**Affiliations:** 1 The Scripps Research Institute, Jupiter, USA

Fanti et al. evaluated twenty commercial antibodies against huntingtin for Western blot, immunoprecipitation, and immunofluorescence by employing a defined experimental approach that involved comparing results in deletion cell lines with isogenic parental controls. This study is a crucial guide that will significantly enhance research on huntingtin biology and Huntington's disease.

 The authors employed twenty commercial antibodies and three distinct cell lines, validating the antibodies across several assays. I have a question concerning IP.

 It is unclear whether the authors employed control beads as an extra control measure during the immunoprecipitation process. Figure 3 necessitates either control beads or immunoprecipitation conducted in knockout cells.

Are sufficient details of methods and materials provided to allow replication by others?

Yes

Is the rationale for creating the dataset(s) clearly described?

Yes

Are the datasets clearly presented in a useable and accessible format?

Yes

Are the protocols appropriate and is the work technically sound?

Yes

Reviewer Expertise:

Mechanisms of neurodegenerative disease

I confirm that I have read this submission and believe that I have an appropriate level of expertise to confirm that it is of an acceptable scientific standard, however I have significant reservations, as outlined above.

# Reviewer report: Reviewer response for version 2

**DOI:** 10.5256/f1000research.176211.r360585

**Published:** 2025-02-06

**Authors:** Hexige Saiyin

**Affiliations:** 1 Fudan University, Shanghai, China

This well-designed study validates twenty HTT antibodies by WB and IF. Based on the reviewer's experience and other studies, HTT is unstable after fixation in human tissue and cells (Ferrante, Gutekunst et al. 1997)(ref 2). Thus, the reviewer suggests providing the details of the fixation in IF and processing the WB sample in WB, especially the staining/blotting time after the fixation or harvesting WB (such as the staining performed immediately after fixation or 48 hours after fixation et al.). 

 Minor concerns:

1. The resolution of IF images is low. The cellular distribution patterns of HTT in cells are difficult to read. Thus, the reviewer encourages showing high-resolution images in IF, which can provide more detail of cellular distribution patterns.
2. HTTs are easy to aggregate (DiFiglia, Sapp et al. 1997, Gutekunst, Li et al. 1999)(ref 1 and 3). The author only detected the single HTT but not the aggregate. Does the antibody that recognizes the single HTT  also equally detect the aggregate of cells or tissue?  Does the author try to detect the aggregate by those antibodies?

Are sufficient details of methods and materials provided to allow replication by others?

Partly

Is the rationale for creating the dataset(s) clearly described?

Yes

Are the datasets clearly presented in a useable and accessible format?

Yes

Are the protocols appropriate and is the work technically sound?

Partly

Reviewer Expertise:

Pathogenesis of HD and 3D histology and pathology of human disease.

I confirm that I have read this submission and believe that I have an appropriate level of expertise to confirm that it is of an acceptable scientific standard.

## References

[1] : Aggregation of huntingtin in neuronal intranuclear inclusions and dystrophic neurites in brain. *Science* .1997;277(5334) : 10.1126/science.277.5334.1990 1990-3 10.1126/science.277.5334.1990 9302293

[2] : Heterogeneous topographic and cellular distribution of huntingtin expression in the normal human neostriatum. *J Neurosci* .1997;17(9) : 10.1523/JNEUROSCI.17-09-03052.1997 3052-63 10.1523/JNEUROSCI.17-09-03052.1997 9096140 PMC6573626

[3] : Nuclear and neuropil aggregates in Huntington's disease: relationship to neuropathology. *J Neurosci* .1999;19(7) : 10.1523/JNEUROSCI.19-07-02522.1999 2522-34 10.1523/JNEUROSCI.19-07-02522.1999 10087066 PMC6786077

# Reviewer report: Reviewer response for version 2

**DOI:** 10.5256/f1000research.176211.r360584

**Published:** 2025-02-05

**Authors:** Christian Landles

**Affiliations:** 1 University College London, London, UK

This manuscript by Fanti *et al. *has comprehensively characterized twenty commercially available HTT antibodies using cell lines which were either wild-type or knock-out for the *HTT* gene. HTT antibody suitability and sensitivity was assessed using standardized experimental protocols using the applications of western blot, immunoprecipitation, and immunofluorescence. This experimental approach was rationally conceived and executed, and the experimental dataset has been presented in a clear manner which was easy to follow and interpret. Overall, this data will be of great use should anyone within the Huntington’s disease research community wish to source and try any specific HTT antibody for their own research needs.

Minor Comments and Recommendations

 Introduction:

 The expression level of the full-length HTT protein is not particularly “low” in brain tissues, it is peripheral tissues which express full-length HTT and low levels.

 Results and discussion:

1. Table 1, it would be useful to report what lineage these cell lines used are from, and also state the CAG repeat size of these cell lines.
2. The MW1 to MW7 antibodies, originally characterized in Ko, *et al*., Brain Res. Bull., 2001, predominantly recognise mutant HTT isoforms where the pathogenic CAG tract has been expanded. Therefore, although stated in the figure legend of Fig. 1 “MW1-S*, MW3-S*, MW4-S*, MW5-S*, MW6-S*, MW7-S* and MW8-S* are expected to only recognize an altered conformation of the polyQ domain generated as the polyQ domain of Huntingtin increases in length.”, I am not sure why these antibodies were being tested here on wild-type DMS 53 cell lines where there was no expanded CAG mutation. A negative signal here was always to be expected.
3. The MW8 antibody, originally characterized in Ko, *et al*., Brain Res. Bull., 2001 [ref 1], and later mapped by Landles *et al*., JBC, 2010 [ref 2], has been demonstrated to be a neo-epitope antibody specifically recognizing the highly pathogenic mutant HTT1a protein isoform. It has subsequently been shown in numerous research papers to not recognise the full-length HTT protein isoform, and so likewise, a negative signal here on wild-type DMS 53 cells was always to be expected. (It would be helpful if these two points were explained fully in the main body of text and not buried in the figure legend of Fig. 1).
4. Since Fanti *et al. *was comprehensively characterizing commercial HTT antibodies, I was very surprised that the commercial monoclonal HTT-MAB5490 and HTT-MAB2166 antibodies were used in their analyses. These two monoclonal HTT antibodies are widely used throughout the entire Huntington’s disease research community, and characterising these here (maybe instead of the MW1-MW8 series) would have been more appropriate / valuable.

Methods:

 In the interest of having all the methodologies used in this already concise manuscript, it would be helpful to the reader to have all the experimental methodologies within this section, as opposed to viewing them from another link online.

Are sufficient details of methods and materials provided to allow replication by others?

Partly

Is the rationale for creating the dataset(s) clearly described?

Yes

Are the datasets clearly presented in a useable and accessible format?

Yes

Are the protocols appropriate and is the work technically sound?

Yes

Reviewer Expertise:

The molecular pathogenesis of Huntingtin's disease, pre-clinical Huntington's disease mouse models, HTT protein bioassays, Molecular biology, HTT protein aggregation.

I confirm that I have read this submission and believe that I have an appropriate level of expertise to confirm that it is of an acceptable scientific standard.

## References

[1] : New anti-huntingtin monoclonal antibodies: implications for huntingtin conformation and its binding proteins. *Brain Res Bull* .56(3-4) : 10.1016/s0361-9230(01)00599-8 319-29 10.1016/s0361-9230(01)00599-8 11719267

[2] : Proteolysis of mutant huntingtin produces an exon 1 fragment that accumulates as an aggregated protein in neuronal nuclei in Huntington disease. *J Biol Chem* .2010;285(12) : 10.1074/jbc.M109.075028 8808-23 10.1074/jbc.M109.075028 20086007 PMC2838303

# Reviewer report: Reviewer response for version 1

**DOI:** 10.5256/f1000research.168592.r315013

**Published:** 2024-09-09

**Authors:** Karen Bowman

**Affiliations:** 1 University of Leicester, Leicester, England, UK

The article pointed out the current technical difficulties in studying Huntington’s Disease and identified the differences in antibodies to the protein Huntingtin, used throughout the HD research community, as a particular problem. The study formed part of the wider YCharOS collaboration to characterise commercial antibodies for human proteins using standardized protocols with the intention of improving antibody reproducibility issues. The study was an evaluation of twenty commercial antibodies for the protein Huntingtin for use in western blot, immunoprecipitation, and immunofluorescence.

 The study was well designed and the results were to a high technical standard and quality. The data is presented clearly and looks robust.

 It was reassuring to see that a committee of industry and academic representatives had endorsed the platform used. The platform consisted of identification of human cell lines suitable for antibody characterisation studies i.e. with adequate levels of Huntingtin, followed by the development of and contribution of isogenic knockout control cell lines, which were validated at the protein level. The final step was a series of antibody characterisation procedures, limited to the most common research uses of antibodies. Limitations of the study are clearly stated.

 Two minor errors were spotted. There is an inconsistency between the text and figure 3, where they mention IP was done on twenty antibodies in the text, but results for only ten antibodies are presented in the figure. Perhaps some additional text on why only ten were presented would be of use, or amend the text to reflect what is presented in the figure. A second error is that in the final results paragraph on immunofluorescence, figure 3 is cited, and linked back to, instead of figure 4.

 Overall, this will be a highly useful resource for scientists in the HD research community.

Are sufficient details of methods and materials provided to allow replication by others?

Yes

Is the rationale for creating the dataset(s) clearly described?

Yes

Are the datasets clearly presented in a useable and accessible format?

Yes

Are the protocols appropriate and is the work technically sound?

Yes

Reviewer Expertise:

drug discovery and diagnostics

I confirm that I have read this submission and believe that I have an appropriate level of expertise to confirm that it is of an acceptable scientific standard.

# Reviewer report: Reviewer response for version 1

**DOI:** 10.5256/f1000research.168592.r315014

**Published:** 2024-08-28

**Authors:** Jieya Shao

**Affiliations:** 1 Washington University in St Louis, St. Louis, Missouri, USA

In this study, the authors performed comprehensive analysis of twenty commercially available HTT antibodies for their suitability and performance in Western blot, immunoprecipitation, and immunofluorescence staining applications. The experimental approaches were logically and thoroughly designed. Data are of high-quality and clearly presented. The results will be very helpful for researchers in the HTT field. However, there seems to be two small errors.

 First, in Figure 3, only the IP data of 10 antibodies were shown while in the text it was stated that 20 antibodies were analyzed.

 Second, on Page 5, in the last sentence of the second paragraph, the authors seem to cite Figure 3 by mistake.

Are sufficient details of methods and materials provided to allow replication by others?

Yes

Is the rationale for creating the dataset(s) clearly described?

Yes

Are the datasets clearly presented in a useable and accessible format?

Yes

Are the protocols appropriate and is the work technically sound?

Yes

Reviewer Expertise:

Cell biology

I confirm that I have read this submission and believe that I have an appropriate level of expertise to confirm that it is of an acceptable scientific standard.
